# Characterizing root response phenotypes by neural network analysis

**DOI:** 10.1093/jxb/erv235

**Published:** 2015-05-26

**Authors:** Sarah V. Hatzig, Sarah Schiessl, Andreas Stahl, Rod J. Snowdon

**Affiliations:** Department of Plant Breeding, IFZ Research Centre for Biosystems, Land Use and Nutrition, Justus Liebig University, Heinrich-Buff-Ring 26–32, 35392 Giessen, Germany

**Keywords:** *Brassica napus*, drought resistance, lateral roots, phenotyping, root architecture, Sholl method.

## Abstract

Roots play an immediate role as the interface for water acquisition. To improve sustainability in low-water environments, breeders of major crops must therefore pay closer attention to advantageous root phenotypes; however, the complexity of root architecture in response to stress can be difficult to quantify. Here, the Sholl method, an established technique from neurobiology used for the characterization of neural network anatomy, was adapted to more adequately describe root responses to osmotic stress. This method was used to investigate the influence of *in vitro* osmotic stress on early root architecture and distribution in drought-resistant and -susceptible genotypes of winter oilseed rape. Interactive changes in root architecture can be easily captured by individual intersection profiles generated by Sholl analysis. Validation using manual measurements confirmed that the number of lateral roots decreased, while mean lateral root length was enhanced, under osmotic stress conditions. Both genotypes reacted to osmotic stress with a shift in their intersection patterns measured with Sholl analysis. Changes in interactive root architecture and distribution under stress were more pronounced in the drought-resistant genotype, indicating that these changes may contribute to drought resistance under mild osmotic stress conditions. The Sholl methodology is presented as a promising tool for selection of cultivars with advantageous root phenotypes under osmotic stress conditions.

## Highlight

The Sholl method, an old, established neurobiology technique for neural network analysis, is ideal for characterization and differentiation of interactive plant root responses to stress.

## Introduction

This paper presents a novel and practicable method for the characterization of plant root architectural properties. The method was adapted from the analysis technique of Sholl (1953), an established neurobiological approach still applied today for morphological characterization of neural networks (Binley *et al.*, 2014; Ferreira *et al.*, 2014; Garcia-Segura and Perez-Marquez, 2014). Known as the Sholl method, this approach uses a coordinate system consisting of a series of concentric circles centred at the soma of a neuron. Local variables are extracted by counting the number of intersections on regular concentric circles surrounding cell dendrites, giving a metric representation of the architectural characteristics of the network and how they change with distance and time. Because roots have similarly dynamic spatial and developmental characteristics to those of a neural network, the Sholl method was tested for its ability to describe topological differentiation in root distribution responses to abiotic stress.

Several climate reports and modelling studies predict a future increase of flooding events in northern Europe, while an increase of drought events is forecast for southern and southeast European countries (Lehner *et al.*, 2006; Alcamo *et al.*, 2007; Bates *et al.*, 2008). While precipitation during winter months is expected to increase slightly, the risk for drought events during the summer cropping season is expected to rise in many areas of Europe (Alderlieste and van Lahnen, 2013). In major agricultural areas of North and South America, Asia, and Australia, crop production is already increasingly threatened by drought. Breeding of cultivars adapted to a broad range of environmental conditions and distinctive annual changes in precipitation are therefore of overriding importance and drought resistance is essential for future crop breeding. *Brassica* napus (oilseed rape) is a major international crop produced in diverse eco-geographical regions and a model polyploid for study of crop evolution and adaptation (Chalhoub *et al.*, 2014).

In a previous study, different winter oilseed rape genotypes were tested in a climate chamber for their *in vitro* osmotic stress responses during a short-term stress period (Hatzig *et al.*, 2014). Drought-resistant genotypes were able to maintain shoot growth under stress conditions, whereas drought-sensitive genotypes reacted with a strong decrease in shoot fresh and dry weight. During different replications of the *in vitro* experiment, one drought-resistant genotype appeared to respond to drought stress by additionally modifying its root architecture; however, differences in fresh or dry weight could not be significantly confirmed. Thus, a need arose for a more sophisticated root phenotyping tool that is able to illuminate potentially beneficial changes in root architecture associated with (*in vitro*) drought stress. Several root evaluation techniques exist for the investigation of root growth characteristics. Many of these also allow a manual or semi-automated analysis of root zone-specific responses [for example, RootReader2D (Clark *et al.*, 2013), RootTrace (French *et al.*, 2009), or WinRhizo (Arsenault *et al.*, 1995)]. However, these methods do not completely capture the interactive architectural properties of the root system as a whole. Here, an easily manageable root phenotyping method, based on the principles of Sholl analysis, was applied for the investigation of root spatial distribution and architecture and their responses to abiotic stress *in vitro*. As far as could be ascertained, this is the first time that this established neurobiological method has been adopted for characterization of plant roots.

## Materials and methods

### Plant materials and cultivation

Two winter oilseed rape (*B. napus* L.) genotypes with different physiological and morphological responses to drought stress were tested for their root responses to osmotic stress during the seedling stage, applying the hydroponic cultivation system as described by Hatzig *et al.* (2014). The two genotypes ‘Ferdie’ (Monsanto Saaten, Nienstädt, Germany) and ‘NPZ 208/03’ (Norddeutsche Pflanzenzucht H.G. Lembke, Hohenlieth, Germany) were classified as drought resistant (DR) and drought sensitive (DS), respectively, corresponding to their drought compatibility (Hatzig *et al.*, 2014). Seedlings were cultivated hydroponically for 22 days in a climate chamber as follows: seeds were sterilised in 3% NaOCl solution for 15min on a shaker. To reduce the surface tension a drop of dishwasher detergent was added. Subsequently the seeds were washed five times in distilled H_2_O and prepared for germination in 0.2mL plastic vessels filled with agar gel [1.5% (w/v)]. One sterile seed per vessel was placed at 1mm depth in the gel matrix. The lower ends of the vessels were cut off so that the roots could reach the nutrient solution in a later stage of the experiment. The vessels were put into a bracket and wrapped in aluminium foil so that germination took place in the dark. After 3 days the seedlings were transferred into 50mL falcon tubes containing MS medium [1.1g L-1 (Murashige and Skoog, 1962)]. Further plant development proceeded in a fully controlled growth chamber under the following conditions: Day period: 16h, 20°C, 65% relative humidity; night period: 8h, 15°C, 65% relative humidity. After 7 days the nutrient solution was changed and nutrient concentration was increased up to 2.2g MS powder per litre.

Each genotype was cultivated under control conditions and under simulated drought stress conditions, with stress treatment commencing on day 17. The plants were transferred into 10L plastic bins (52cm × 12cm × 13cm) containing fully concentrated nutrient solution (4.3g MS powder per L). For the stress treatment 2.5% PEG 6000 was added to the hydroponic medium on day 17, with the PEG concentration (w/w) being increased to 5% after a further 48h. The corresponding osmotic potentials can be calculated as follows using the formula of Michel and Kaufmann (1973): −0.022MPa at 2.5% PEG 6000 and −0.058MPa at 5% PEG 6000. To ensure oxygen supply the solutions were constantly aerated using an air pump. After 3 days at 5% PEG the roots of control and stressed plants were harvested for image analysis.

### Root evaluations and imaging

Roots were placed for 5min on an absorbent paper and fresh weights (FW) were subsequently determined using a fine scale. The roots were placed on a glass scanner plate and disentangled by pipetting a thin layer of water onto the root body. Roots were scanned with a common office scanner at a resolution of 200 dpi. For each genotype and treatment five biological replicates were imaged. Significant differences were calculated with pairwise t-test (*P* < 0.1) in the R statistical software package (R Development Core Team, 2008), version 3.0.2. Compliance with normal distribution and homogeneity of variances were evaluated using a Bartlett test. If variances were homogeneous, significances were calculated using a pairwise t-test with var.equal = TRUE. For datasets with inhomogeneous variances, a pairwise t-test with var.equal = FALSE was implemented.

**Fig. 1.**
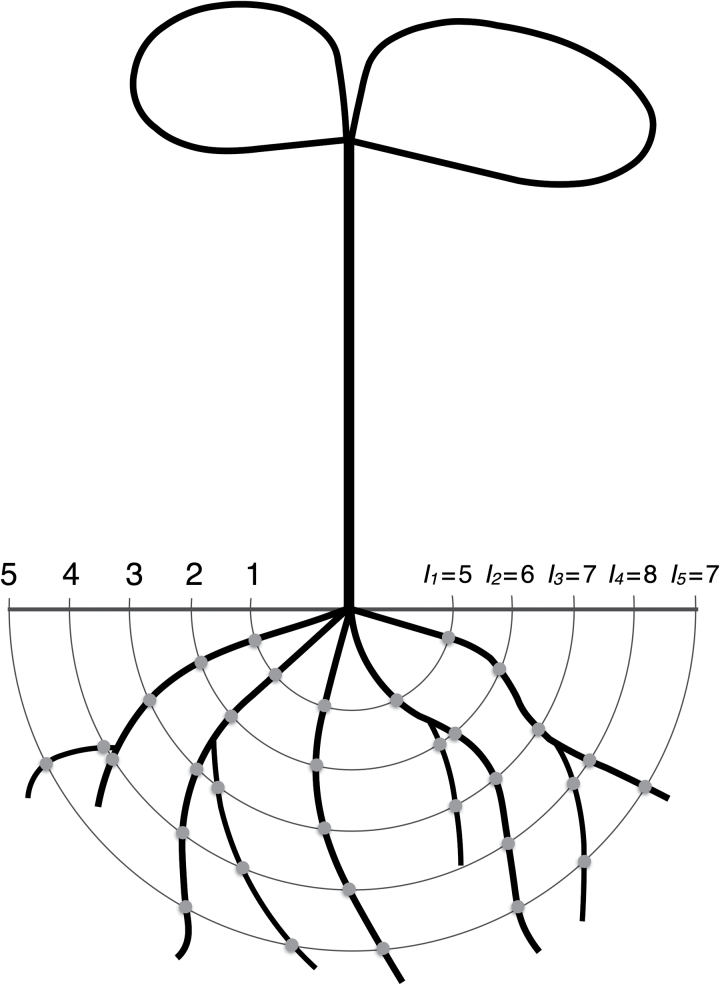
Principle of root phenotyping by Sholl analysis, in which concentric circles are drawn at regular intervals around the root origin and the number of root-circle intersections was counted for each circle (this figure is available in colour at *JXB* online).

Total root length (RL), primary root length (PRL), and lateral root length (LRL) were measured manually by tracing the roots with the freehand line tool of the software ImageJ (rsb.info.nih.gov/ij/). Mean length of lateral roots (MLRL) was calculated. The number of lateral roots (NLR) was determined by manual counting. Concentric circles were drawn with a common compass around the root origin (Fig. 1) at intervals of 0.5cm. The first circle was drawn at a distance of 0.5cm from root origin and the outer circle was drawn beyond the outermost root tip.

### Sholl analysis

Sholl analysis was performed with the software ImageJ using the plugin Sholl analysis (version 3.0). Before starting the image analysis, the root pictures were converted into a 16 bit threshold greyscale image and the pixel distance was converted into a known distance with the ‘Set scale’ function accessible in the ‘Analyze’ menu. After brightness adjustment and manual setting of the root origin, Sholl analysis was started. The linear method was selected, in which the starting radius, radius step size, and ending radius are chosen by the user. Concentric circles were then drawn automatically at regular intervals around the root (Fig. 1). According to the growth characteristics of the *B. napus* seedling roots used as a model, a circle interval of 0.5cm was chosen, with a starting radius of 0.5cm for the innermost circle and an outer radius beyond the outermost root tip. A further decrease in circle intervals did not lead to an additional gain of information. Sholl analysis determines the architectural characteristics of a network by documenting the number of root-circle intersections in relation to their distance from the centre of origin. After parameter setting, the counting of intersections per radius occurs automatically. Depending on the chosen radius step size, the analysis takes up to a few seconds. For specification of root characteristics, two parameters were evaluated. The first was the absolute number of root-circle intersections, defined as the sum of root intersections of all concentric circles (*I_T_*). This number can be represented by the following formula:

$$I_{T}=\sum_{k=1}^{n}I_{k}$$

where *I_k_* is the count of intersections of circle *k*.

Furthermore, the circle-specific numbers of intersections were plotted against the corresponding circle radii, in order to gain information about the number of roots located at a specific distance to the root origin. To evaluate the effect of the root spreading on the positional data delivered by the Sholl analysis, the winter oilseed rape line ‘Adriana’ (Limagrain, Germany) was cultivated in pots (11×11×12cm) filled with sand for 22 days in the greenhouse under semi-controlled conditions. The washed root system from a single plant was scanned 10 times independently, with repeated rinsing and re-spreading of the roots each time.

### Data validation via manual counts

The values from manual counting of circle-specific intersections and the automated counting by Sholl analysis were correlated separately for each biological replication. Pearson’s product moment correlation was then calculated after Fisher’s z-transformation. Principal component analysis (PCA) was carried out with results from Sholl analysis (circle-specific intersection patterns) and first components were then correlated with the manual measurements of root traits (RL, NLR, PRL, LRL, MLRL) using Pearson’s product moment correlation.

## Results

**Fig. 2.**
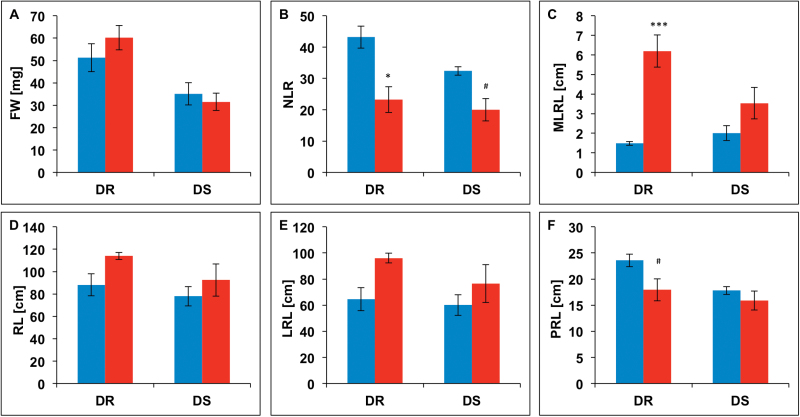
Effect of *in vitro* osmotic stress on (A) root fresh weights (FW), (B) the absolute number of lateral roots (NLR), (C) the mean length of lateral roots (MLRL), (D) the total root length (RL), (E) the total length of lateral roots (LRL), and (F) the length of the primary root (PRL) of a DR and a DS winter oilseed rape genotype, measured under control conditions (left bars) and after 5 days of PEG 6000 treatment (right bars). Bars show mean measurements of five replicate plants with standard errors indicated by whiskers. Significant differences at ^#^ *P* < 0.1, *P < 0.05, **P < 0.01 and ***P < 0.001 (this figure is available in colour at *JXB* online).

For both genotypes no significant differences in root FWs could be observed between control and PEG 6000 treatment (Fig. 2A). For genotype DR the mean root FW was 51.2mg in the control treatment and 60.2mg in the PEG 6000 treatment. For genotype DS a mean root FW of 35.1mg was measured in the control treatment, with 31.5 in the PEG 6000 treatment. Under osmotic stress conditions, NLR decreased significantly in both genotypes (Fig. 2B). In genotype DR, NLR decreased to almost 50% compared to the control, while genotype DS showed a reduction of 40% under stress. In contrast, osmotic stress caused an increase in MLRL in both genotypes. However, significant differences between the treatments could be confirmed for genotype DR only, which showed an increase from 1.48cm (control) to 6.19cm (PEG 6000) (Fig. 2C). Mean values for RL and LRL in both genotypes were increased under stress but not significantly different from the measurements in the control treatment (Fig. 2D, E). PRL was significantly reduced under osmotic stress in genotype DR, while no differences could be observed for DS (Fig. 2F).

### Sholl analysis reveals differentiation of root branching characteristics

**Fig. 3.**
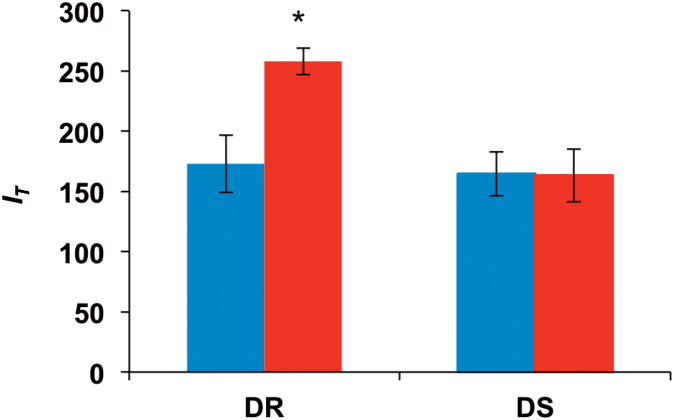
Effect of *in vitro* osmotic stress on the absolute number of intersections measured with Sholl analysis of a DR and a DS winter oilseed rape genotype, measured under control conditions (left bars) and after 5 days of PEG 6000 treatment (right bars). Significant differences at *P < 0.05 (this figure is available in colour at *JXB* online).

**Fig. 4.**
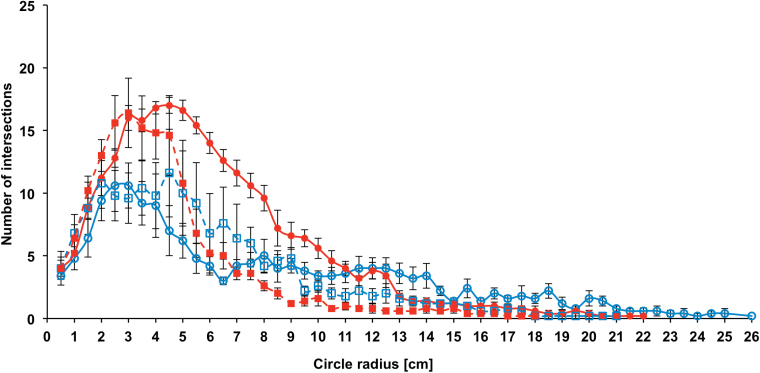
Number of root intersections depending on the distance from root origin of a DR (dotted lines) and a DS (squared lines) winter oilseed rape genotype, measured under control conditions (open symbols) and after 5 days of PEG 6000 treatment (closed symbols). Points are means of five replicates with standard errors (this figure is available in colour at *JXB* online).

**Fig. 5.**
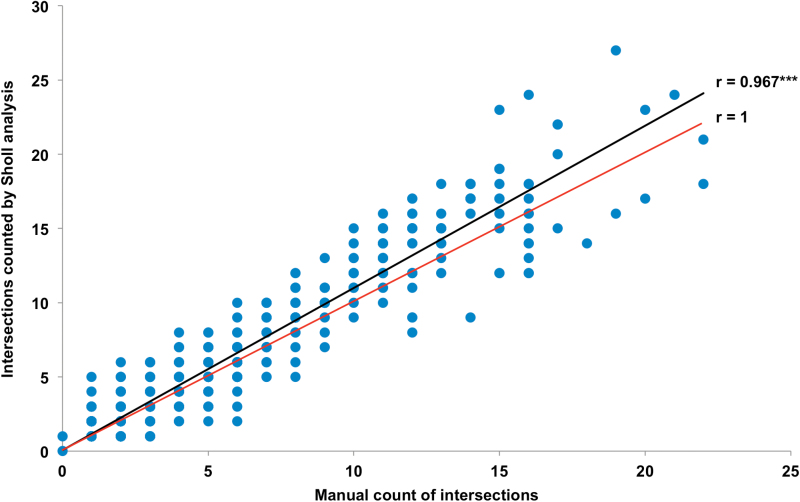
Correlations between the manually counted number of intersections and the number of intersections measured with Sholl analysis. Measurements were done on roots of two different winter oilseed rape genotypes cultivated under control and osmotic stress conditions. Regression lines were indicated for the calculated correlation (r = 0.967) and the maximum possible correlation (r = 1) between manually counted intersections and intersections counted by Sholl analysis. Total sample size: n = 20. Significance at ***P < 0.001 (this figure is available in colour at *JXB* online).

No difference in absolute number of root-circle intersections was observed between the control treatments of the two investigated *B. napus* genotypes (Fig. 3). Under osmotic stress conditions, however, genotype DR reacted with a significant increase in intersection number, while for genotype DS no difference in intersection number could be observed between treatments. In genotype DR the absolute number of intersections increased from 173 (control) to 258 (PEG 6000), while DS showed 165 intersections under control conditions and 163 after PEG 6000 treatment. Under control conditions the last root-circle intersection in genotype DR occurred at a radius of 26cm, whereas for genotype DS the farthest circle intersection was measured at 21cm from the root origin (Fig. 4).Outermost root intersection under PEG 6000 treatment was measured at radii of 22cm and 18cm for DR and DS, respectively. Under control conditions, both genotypes showed a similar number of root-circle intersections between 0.5 and 4cm distance to the root origin. The number of root intersections was significantly lower at 6.5cm for genotype DR than for DS, whereas it was significantly higher at most circles between 9.5 and 26cm from the origin. Both genotypes showed a significant circle-specific increase in intersection number in the PEG 6000 treatment. In DR the intersection number increased significantly between 3 and 10cm, whereas DS showed significantly higher intersection numbers from 2.5 to 3cm from the origin. Intersection number in DS dropped significantly below that of the control treatment between 7.5 and 9cm, while no differences between treatments could be observed between 9.5 and 18cm. For DR the intersection number between 13 and 21cm was almost always significantly lower in the PEG 6000 treatment than that of control treatment. A very strong correlation (r > 0.967) was observed between the manual counting of circle-specific root intersections and the automated counting by Sholl analysis (Fig. 5).

### Correlations among root traits

PRL was significantly correlated with FW and NLR. LRL showed a very strong correlation with RL (r > 0.9) (Table 1). For MLRL significant correlations with NLR, RL, PRL, and LRL were calculated. The absolute number of intersections measured with Sholl analysis showed significant correlations of r > 0.5 with the traits FW, RL, LRL, and MLRL. All manually measured root traits (NLR, MLRL, RL, PRL, and LRL) showed significant correlations with the first two components of the PCA, including circle-specific intersection patterns of Sholl analysis (Table 2).

**Table 1.** Correlations between root parameters and total number of root-circle intersections measured with Sholl analysis

	FW	NLR	RL	PRL	LRL	MLRL
NLR	0.10					
RL	0.31	−0.16				
PRL	0.41^#^	0.84***	0.11			
LRL	0.24	−0.31	0.98***	−0.07		
MLRL	0.27	−0.71***	0.69***	−0.48*	0.77***	
IT	0.56**	0.00	0.67**	0.23	0.63**	0.54*

FW, fresh weight; LRL, lateral root length; MLRL, mean length of lateral roots; NLR, absolute number of lateral roots; PRL, primary root length; RL, total root length. Total sample size: n = 20. Significances at ^#^0.1, *P < 0.05, **P < 0.01, and ***P < 0.001.

**Table 2.** Correlations between the first five components of a PCA including values from Sholl analysis and manually measured root traits

	PC1Sholl (37.4%)	PC2Sholl (20.6%)	PC3Sholl (11.1%)	PC4Sholl (7.7%)	PC5Sholl (5.7%)
NLR	0.69***	0.42#	0.18	−0.15	0.09
MLRL	−0.14	−0.77***	-0.25	0.25	0.04
RL	0.32	−0.56**	0.13	0.05	0.13
PRL	0.79***	0.16	0.09	−0.33	−0.06
LRL	0.18	−0.59**	0.12	0.11	0.14

LRL, lateral root length; MLRL, mean length of lateral roots; NLR, absolute number of lateral roots; PCSholl, PCA including values from Sholl analysis; PRL, primary root length; RL, total root length. Total sample size: n = 20. Significances at ^#^0.1, *P < 0.05, **P < 0.01, and ***P < 0.001.

## Discussion

### Interactive root responses to osmotic stress

Water deprivation has a manifold nature, with its impact *inter alia* being strongly dependent on the drought pattern (Fischer and Turner, 1978) and seasonal timing (Richards, 1996). On the other hand, plant reactions in terms of architecture and physiology are also multifarious. Generally, changes in root topology are connected with maintenance of the hydraulic status under drought conditions (Comas *et al.*, 2013). A frequently observed phenomenon is that plants adopt physiological strategies to maintain root growth during times of water scarcity. Osmotic adjustment is shown to be a suitable tool for the maintenance of growth effective turgor pressure in the root elongation zones (Voetberg and Sharp, 1991). However, the nature of root architectural responses to drought is strongly dependent on the species, stress shaping, and soil conditions (Bengough *et al.*, 2006; Ito *et al.*, 2006). Any particular part of the root system has a highly specific physiological age that can differ strongly from other roots on the same plant. Hence the phenotypic variation along the length and breadth of the root system reflects not only differences in root physiology and responsiveness, but also the range of genotype-by-environment interactions to which the plant was exposed following germination (Bengough *et al.*, 2006; Forde, 2009). The results demonstrated in this study suggest that osmotic stress in winter oilseed rape invokes an increase in the mean length of lateral roots as an early adaptive response. The 4-fold higher MLRL observed in a genotype resistant to drought under osmotic stress (Fig. 2C) was not observed in the drought-sensitive genotype and was only seen as a trend under stress conditions. However, this apparently drastic response contributed neither to a significant increase of RL nor of total LRL, as it occurs at the expense of the absolute NLR in both genotypes. A reduction in the NLR was shown to be correlated with a decrease in the PRL, a phenomenon that was observed in the stress treatment as well (Table 1).

A stimulating effect of drought on lateral root length has previously been observed (Jupp and Newman, 1987; van der Weele *et al.*, 2000; Yang *et al.*, 2004). However, different effects of drought were observed for the number of lateral roots. On the one hand, a reduction in the number of lateral roots, as found in the present study, was confirmed by other studies (van der Weele *et al.*, 2000; Placido *et al.*, 2013). On the other hand, drought can also cause an increase in the number of lateral roots (Jupp and Newman, 1987; Yang *et al.*, 2004). The coherence between drought compatibility and root plasticity is a controversial topic. Xiong *et al.* (2006) provided evidence for an inhibitory effect of drought stress on lateral root formation in *Arabidopsis thaliana* and concluded that the formation of longer vertical roots, reaching deeper soil layers, is more advantageous for water uptake. While this assumption probably holds true for certain climatic scenarios and soil conditions, it cannot be regarded as a general rule. In contrast, Ehdaie *et al.* (2012) showed that an increase in lateral root formation in *Triticum aestivum* (bread wheat) was associated with increased yield stability under certain forms of drought. Although a decrease in the number and an increase in the mean length of lateral roots under osmotic stress is not a commonly observed phenomenon, the present results show that this seems to be an adaptive response of young rapeseed plants to mild osmotic stress. Furthermore, osmotic stress causes a shift in circle-specific intersection patterns of both genotypes, indicating changes in root spatial distribution (Fig. 4). The stronger reaction in both the absolute number of root-circle intersections (Fig. 3) and the number of circle-specific intersections under osmotic stress in the DR genotype compared to the DS genotype (Fig. 4), suggest that root architectural changes and changes in root distribution are more distinct in DR and consequently lead to better drought compatibility, while architectural changes in DS are not sufficient to impart drought resistance.

### Sholl analysis quantifies root architectural plasticity

**Fig. 6.**
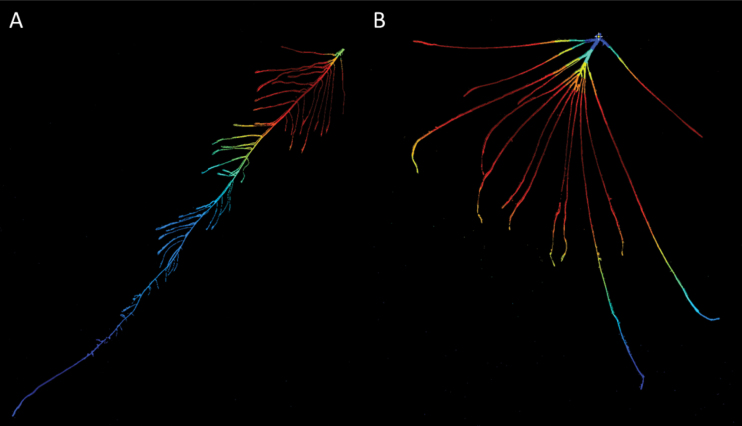
Examples for Sholl’s intersection masks of two contrasting extreme winter oilseed rape root phenotypes A (DR, control) and B (DR, PEG 6000).

As demonstrated in the examples presented here, Sholl analysis helps to capture the interactive architectural properties of an entire root system and its response to the environment (Fig. 6). The Sholl parameters assessed revealed different root architectural and distributional responses to osmotic stress in two contrasting genotypes that seem to be involved in the manifestation of drought compatibility in *B. napus*. The results of the Sholl analysis suggest that root architectural characteristics are significantly influenced by osmotic stress even when such changes are not necessarily obvious from root biomass or RL (Fig. 2A,D). The absolute number of intersections captured depends particularly on the RL and on LRL and MLRL (Table 1). Sholl analysis revealed a significant increase in the absolute number of root-circle intersections in the drought-resistant genotype under osmotic stress, whereas the drought-sensitive genotype did not show a corresponding response (Fig. 3). This indicates that genotype DR has a greater reaction in the above-mentioned root properties than DS, likely conferring effective adaptation and growth maintenance under the given stress conditions.

A very interesting insight into the changes of root architecture and distribution was given by the spatial values provided by Sholl measurements. Different distance-specific intersection patterns were generated depending on the genotype and the treatment (Fig. 4). These patterns were converted into principal components that sowed marked correlations to all manually measured root traits (Table 2). It is thereby evident that Sholl analysis is a phenotyping tool suitable for an interactive discovery of root architectural properties such as the root length, the number of roots, and their spatial arrangement. Especially the latter could also be described by the absolute values of the distance-specific intersection patterns. In contrast to root density, which captures the cumulative number of roots per volume or area, Sholl analysis captures the absolute value of roots, located at a specific distance to the root origin. From the patterns shown in Fig. 4, it can be concluded that osmotic stress in oilseed rape enhances the number of roots present in the upper half of the root, while it causes a reduction in the number of roots towards the root tip.

### Advances and limitations of Sholl analysis for root characterization

**Fig. 7.**
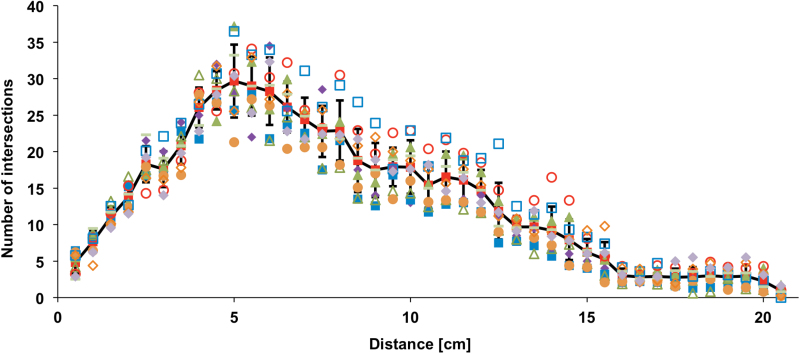
Number of root intersections depending on the distance from root origin of a single soil grown winter oilseed rape root, repeated in 10 technical replications by re-spreading the root 10 times on the scanner plate. Sholl analysis was repeated on each scan. The single replications are represented by different symbols. Mean values are represented by a solid line. Error bars represent standard deviations (this figure is available in colour at *JXB* online).

Sholl analysis is a very simple and non-destructive method for characterization of root architecture and distribution in plants grown under hydroponic cultures. It is easy to apply Sholl analysis using the free, open-source software ImageJ. As the Sholl method relies on punctual measurements, it does not require the extremely high image quality and resolution necessary for measurements of root longitude and growth dynamics. The method could be validated by the manual counting of roots intersecting hand-drawn circles (Fig. 5). The slight overestimation of intersection number in comparison to the manual counts might be caused by sampling errors caused by pixel background noise in the images. These artefacts might be eliminated by an increase in image quality. To evaluate positional effects, the complex root system of a 22-d-old, soil-grown rapeseed plant was placed 10 times individually on the scanner plate. Calculations of standard deviations accounted for slight positional effects, while intersection patterns remain highly similar for all root images (Fig. 7). Positional effects might be excluded by using roots that are fixed in gel-based rhizotrons, for example.

One potential limitation of the Sholl method is the restriction to 2D root properties. Furthermore, Sholl analysis does not differentiate whether changes in intersection number are caused by alterations in root length or by the number of roots. Results could be biased by curved root segments that cross the same circles more than once. Moreover, different root types cannot be distinguished. Therefore, if specific information about these root characteristics is required, the method has to be complemented by other specific software for measuring global root characteristics. A number of such packages provide information on type-specific root number and length or growth dynamics, for example RootReader2D (Clark *et al.*, 2013), RootTrace (French *et al.*, 2009), or WinRhizo (Arsenault *et al.*, 1995). In fact Sholl analysis represents a potentially useful additional tool for commercial root analysis software, because it captures interactive and responsive properties of a root system in a single step. The presented results showed that a significant effect of osmotic stress could be observed by intersection number and pattern, but not in terms of FW or root length characteristics such as RL, LRL, or PRL (except for PRL in genotype DR). This indicates that the Sholl method is more sensitive to mild osmotic stress applications than single biomass or length measurements. These results show that differences in interactive root architecture and spatial arrangement can be reliably investigated in a single time-saving step by applying the principles of Sholl analysis.

## Conclusions

Sholl analysis, an established method for neural network analysis, was shown to be a powerful tool for the disclosure of early interactive root properties in seedlings of winter oilseed rape. This method unravels complex architectural changes, including root length properties and number of roots along with their spatial arrangement, that may be overlooked by singular-dimensional measurements. The method thus provides a novel opportunity for the selection of plant cultivars with beneficial root phenotypes and enhanced drought compatibility. An acceleration of data extraction by semi-automatic root image analysis might form a basis for inclusion of Sholl analysis in high throughput phenotyping platforms. Use of Sholl analysis can potentially provide added power to established root phenotyping software. The usefulness of Sholl analysis for studies investigating the basis of drought resistance in crop plants has been demonstrated here.

## Abbreviations:

DR: drought resistant
DS: drought sensitive
FW: fresh weight
LRL: lateral root length
NaOCl: sodium hypochlorite
MLRL: mean lateral root length
MS: powder, Murashige and Skoog nutrient mixture
NLR: number of lateral roots
PEG: polyethylene glycol
r: radius
PC: principal component
PCA: principal component analysis
PRL: primary root length
RL: total root length
dpi: dots per inch.
